# Improving Strength and Fatigue Resistance in Post-Polio Syndrome Individuals with REAC Neurobiological Treatments

**DOI:** 10.3390/jpm13111536

**Published:** 2023-10-26

**Authors:** Monalisa Pereira Motta, Acary Souza Bulle Oliveira, Jeyce Adrielly André Nogueira, Alcione Aparecida Vieira de Souza Moscardi, Claudete Munhoz Teixeira, Vanessa Manchim Favaro, Amanda Orasmo Simcsik, Salete Conde, Maria Clara Patrizi, Chiara Rinaldi, Vania Fontani, Salvatore Rinaldi

**Affiliations:** 1 Division of Neuromuscular Diseases, Department of Neurology and Neurosurgery, Federal University of São Paulo, São Paulo 01000-000, Brazil; monalisa.motta@gmail.com (M.P.M.); jaanogueira21@unifesp.br (J.A.A.N.); claudetemunhoz71@gmail.com (C.M.T.); v.favaro@unifesp.br (V.M.F.); amanda.simcsik11@gmail.com (A.O.S.); saleteconde@icloud.com (S.C.); clarinhaclarinha27@gmail.com (M.C.P.); 2 Department of Preventive Medicine, Federal University of São Paulo, São Paulo 01000-000, Brazil; moscardi.alcione@unifesp.br; 3 Department of Neuroscience, Psychology, Drug Area, and Child Health (NEUROFARBA), University of Florence, 50134 Florence, Italy; chiara@irf.it; 4 Department of Adaptive Neuro Psycho Physio Pathology and Neuro Psycho Physical Optimization, Rinaldi Fontani Institute, 50144 Florence, Italy; vfontani@irf.it; 5 Department of Regenerative Medicine, Rinaldi Fontani Institute, 50144 Florence, Italy; 6 Research Department, Rinaldi Fontani Foundation, 50144 Florence, Italy

**Keywords:** post-polio syndrome, manual muscle testing, time up and go test, handgrip strength, piper fatigue scale, neurobiological modulation, radio electric asymmetric conveyer

## Abstract

Post-Polio Syndrome (PPS) is a chronic condition characterized by the emergence of new symptoms and functional decline in individuals who previously had polio. Despite advances in medical understanding, management of PPS remains challenging. This study aimed to evaluate the use of neurobiological modulation treatments using Radio Electric Asymmetric Conveyer (REAC) technology on fatigue and muscle strength. An open-label study was conducted with 17 patients submitted to four neuromodulation protocols: Neuro Postural Optimization (NPO), Neuro Psycho Physical Optimization (NPPO), Neuro Psycho Physical Optimization—Cervico Brachial (NPPO-CB), and Neuromuscular Optimization (NMO). The Time Up and Go (TUG) test, Handgrip Strength Test, and Revised Piper Fatigue Scale (RPFS) were used to assess participants’ fatigue and muscle strength, being applied at the beginning and end of each protocol. The results obtained from the improvement in strength, physical endurance, and particularly the RPFS behavioral dimension, affective dimension, and psychological sensory dimension, through the utilization of REAC neurobiological modulation treatments, highlight this correlation. These results suggest that these treatments could be considered as a potential therapeutic approach for PPS.

## 1. Introduction

Post-Polio Syndrome (PPS) is a long-term condition [1] that affects individuals who have previously contracted poliomyelitis (a viral infection primarily impacting the nervous system). The percentage of polio patients affected by PPS varies widely, ranging from 20% to 85% [2,3,4,5,6], depending on the diagnostic criteria utilized [7]. The disease manifests at least 15 years after the initial polio infection [7,8], presenting symptoms, such as muscle weakness, fatigue, muscle pain and or joint pain, cold intolerance, cramps, sleep disorder, reduced functional capacity, thus negatively affecting the quality of life of these individuals [1].

Emerging muscle weakness and fatigue are among the most frequent and debilitating late symptoms of poliomyelitis, causing significant functional impairment in the functional capacity of people with PPS [9,10,11]. Individuals with PPS who experience muscle weakness will need to carry out daily activities with contraction intensities exceeding their maximal capacity. This can cause a progressive reduction in work capacity and lead to uncommon fatigue [12].

The combination of new symptoms and primary polio-related deficits results in significant limitations, and unexpected impairments and/or disabilities can have a psychological impact. These symptoms can be psychologically devastating. Individuals may experience a recurrence of symptoms similar to those they experienced during their acute illness, which they thought they had permanently overcome [13].

The uncertainty and unpredictability of symptom progression in PPS can generate anxiety about the future and concerns regarding the ability to manage the physical and emotional challenges associated with the condition.

Until this day, the treatment of fatigue and muscle strength in PPS is based on lifestyle changes, physiotherapy, training programs, and the prevention of secondary complications [13]. Patients with PPS can be considered a vulnerable population because they are at a disadvantage of having special needs and their lack of therapeutic alternatives. However, it becomes a good condition for the use of this technology since it does not involve risks and discomforts.

In this sense, it is important to investigate targeted therapeutic strategies that can effectively address the unique challenges faced by individuals with PPS. By focusing on these key aspects, Radio Electric Asymmetric Conveyer (REAC) biotechnology was developed with the intention of offering new therapeutic strategies aimed at recovering the correct mechanisms of neurobiological functions that may have been altered by dysfunctional phenomena of epigenetic adaptation, in particular at the psychophysical level.

One of the notable advantages of REAC treatments is their non-invasiveness and absence of adverse effects, in addition to being painless and quick to apply (from fractions of seconds to a few minutes), making it a safe option for diverse populations. Specifically, this investigation aims to evaluate the effects of the REAC Neuro Postural Optimization (NPO), Neuro Psycho Physical Optimization (NPPO), Neuro Psycho Physical Optimization—Cervico Brachial (NPPO-CB) and Neuromuscular Optimization (NMO) neurobiological treatments on strength, fatigue, and functional capacity in individuals with PPS.

## 2. Materials and Methods

### 2.1. Study Design

Open-label study, conducted in accordance with the Declaration of Helsinki [14] and approved by the ethics committee of Federal University of São Paulo–UNIFESP, protocol code 4.526.882.

### 2.2. Sample Size Determination and Power Analysis

After conducting a sample power analysis using G*Power (Universität Düsseldorf, Psychologie—HHU) [15,16] and setting the following parameters: statistical test Wilcoxon signed-rank test one sided, effect size value of 0.5, alpha error probability of 0.05, and power of 0.5, we determined that a total sample size of 13 subjects was required. In order to address the potential occurrence of participants dropping out, we opted to augment the study’s patient population.

### 2.3. Inclusion and Exclusion Criteria

Due to the great heterogeneity of this population, we aimed to make the studied population as homogeneous as possible.

Ambulatory subjects with PPS were included if they had: confirmed history of poliomyelitis with electroneuromyography; confirmed diagnosis of PPS; new muscle weakness for at least one year; aged between 18 and 65 years, male or female; ability to walk at least three meters with or without additions and orthoses; preserved capacity for verbal communication; ability to understand the information about the study and document their decision about their participation in the study in the free and informed consent form.

The criteria for exclusion were: individuals with a degree of muscle strength less than four were excluded; patients with associated diseases that also lead to motor neuron involvement (motor neuron disease and spinal muscular atrophy), diabetes mellitus, alcoholism, thyroid disease, multifocal conduction block, Multiple Sclerosis, nutritional deficiency, autoimmune diseases, exogenous intoxication (heavy metals and insecticides).

### 2.4. Research Locations

The study was conducted in the Division of Neuromuscular Diseases, Department of Neurology and Neurosurgery, Federal University of São Paulo, São Paulo, Brazil.

### 2.5. Population

The study comprised 17 PPS patients. The treatments (pharmacological or not) prior to the study were maintained according to medical advice.

### 2.6. Procedures and Duration of the Study

Once patients had been recruited in accordance with the inclusion and exclusion criteria and informed consent had been obtained, the study commenced, with patients undergoing four treatment protocols (NPO, NPPO, NPPO-CB, and NMO) divided into different stages. It is important to note that each treatment protocol was different in its application and administration.

T0: Pre-intervention period, all assessments were performed at this time;

T1: Administration of the NPO protocol, which consists of a single treatment of approximately 1 s applied via the metal tip of the REAC probe to a specific point in the external auditory pavilion (ear).

T2: Post-REAC NPO/pre-NPPO assessments: The NPPOs were administered concurrently, with patients receiving the NPPO initially, followed by the NPPO-CB, entailing 18 treatment cycles using radiofrequency pulses. The NPPO protocol was implemented with a metal tip on seven specified points of the auricle. The NPPO-CB procedure involved placing a smooth probe covered by a tissue plate onto the cervicobrachial area, secured with a tubular mesh, and left in position briefly. The NPPO-CB application was administered immediately following each NPPO application. This resulted in eight daily applications (4 NPPO + 4 NPPO-CB) separated by a one-hour interval between each.

T3: Post-REAC NPPOs and Pre-NMO assessments.

The NMO treatment method involves the application of radiofrequency pulses through probes covered by tissue plates that are fixed over the lower limbs’ treated area. The treatment plan consists of 10 applications divided into three cycles. During the first two cycles, four applications were carried out each day, while in the third cycle, there were two applications, with a one-hour interval between them.

T4: Post-REAC NMO assessments.

**Figure 1:**
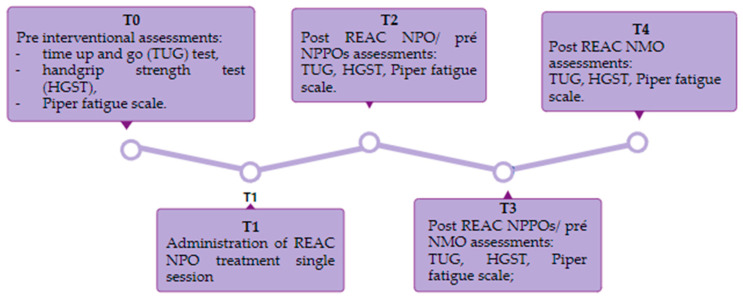
T0: pre-interventional assessments; T1: Administration of REAC NPO; T2: Post-REAC NPO/pre-NPPOs assessments; T3: post-REAC NPPOs/pre-NMO assessments; T4: post-REAC NMO assessments.

During the neuromodulation treatment cycles and the observation phase of REAC-NPPO, no participant received any additional concurrent treatments. A seven-day interval between each protocol was strictly observed. Considering the time demands and application schedule of NPPO, NPPO-CB, and NMOs, the participants were split into two groups with treatments on different days of the week but at the same time in the morning. The protocol spanned a period of 12 weeks, during which assessments and applications were conducted. All evaluations were carried out before and after each stage of the research, as illustrated in Figure 1.

### 2.7. Muscle Strength and Fatigue Testing

#### 2.7.1. Time Up and Go

The TUG is a widely utilized assessment tool for evaluating mobility and functional performance in individuals [17]. It measures the time taken by an individual to complete a series of activities that simulate common daily tasks requiring mobility [18,19]. During the TUG, the individual initiates the test in a seated position on a chair. Upon a verbal cue, they rise from the chair, walk a distance of 3 m, perform a turn, walk back to the chair, and sit down again. The timing starts from the moment the verbal cue is given and stops when the individual is seated back in the chair [17,18].

The TUG yields valuable information about multiple aspects of mobility and functional ability, including balance, gait speed, agility, and the performance of basic mobility tasks. It is particularly beneficial in evaluating individuals who may be at risk of falls or experience mobility limitations due to various conditions, such as aging, neurological disorders, or musculoskeletal impairments [17,18,20].

Interpreting the results of the TUG is based on the time required to complete the test. Generally, shorter completion times indicate better mobility and functional performance, while longer times suggest limitations or difficulties in performing the tasks. However, interpretation may vary depending on the specific population being assessed and the designated cutoff values [18,20,21].

In our study, patients were given prior instructions on how to perform the test and were instructed to walk at their habitual speed and stride. Three measurements were conducted with a one-minute interval, and the best performance (shortest time) was considered as the final measurement.

Time values below 10 s indicate individuals who possess complete freedom and independence. Patients who complete the test within 10 to 19 s are deemed independent, demonstrating reasonable balance and walking speed. Most of them are capable of walking more than 500 m, climbing stairs, and independently leaving their homes. Those who take between 20 and 29 s fall into a “gray area”, signifying challenges with daily tasks that can vary significantly depending on different situations encountered, necessitating good balance, a minimum walking speed of 0.5 m/s, and functional capacity. Subjects with a time score of 30 s or more tend to be fully dependent on many basic and instrumental activities of daily living, such as rising from a chair, self-feeding, changing clothes, bathing, and walking [18,20,21].

#### 2.7.2. Handgrip Strength Test (HGST)

The HGST is a common dynamometry test employed for assessing the maximum force exerted by an individual’s hand and forearm muscles [22]. This test provides valuable information regarding the strength and functionality of these muscle groups [23].

The HGST was conducted using a handheld dynamometer, a device specifically designed to measure grip strength. The dynamometer was adjusted to accommodate the individual’s hand size and was positioned in a manner that allowed a comfortable grip. Instrutherm^®^ manual digital dynamometer was used.

Prior to the test, participants were provided with clear instructions on proper hand placement and technique. They were instructed to hold the dynamometer in their dominant hand or/least affected limb with a firm grip, avoiding excessive squeezing or slackness. The forearm was positioned neutrally, and the arm was extended along the body. Participants were advised to refrain from using their body or non-tested hand for support during the test.

Each participant performed the test three times, with a brief resting period of one minute between trials. During each trial, participants were instructed to exert maximum force while squeezing the dynamometer for a duration of approximately five seconds. The highest recorded grip strength value from the three trials was considered the representative measurement for that hand.

To ensure accurate and consistent measurements, the dynamometer was calibrated before the commencement of the study, following the manufacturer’s guidelines. The researcher administering the test was trained in proper testing procedures to minimize inter-operator variability.

#### 2.7.3. Revised Piper Fatigue Scale (RPFS)

The RPFS [24] is a scientifically validated tool used to assess and quantify fatigue experienced by individuals in a variety of contexts, particularly in the field of healthcare and clinical research. Developed by Dr. Frances C. Piper and her colleagues [24,25], this scale provides a comprehensive assessment of fatigue by considering multiple dimensions and aspects of the experience. The instrument consists of 23 alternative questions and 5 descriptive questions that address the sensory, cognitive, mood, behavioral, severity, and affective aspects of fatigue; distributed in three dimensions: behavioral, affective, and psychological sensory. The patient is given a numerical scale from 0 to 10 as a response option. The score is calculated by averaging all the items in the instrument (items 2 to 23), and the dimension scores are calculated by averaging the items in each dimension. As the items are described on a numerical scale from zero to ten, this means that the higher the score, the greater the fatigue [24,25].

RPFS has been widely used in clinical trials, research studies, and clinical practice to evaluate fatigue levels, track changes over time and assess the effectiveness of interventions aimed at managing or reducing fatigue.

### 2.8. Radio Electric Asymmetric Conveyer (REAC) Technology and Neuro Psycho Physical Optimization Treatments

#### 2.8.1. REAC Technology

The REAC technology is an innovative therapeutic approach that utilizes radio electric fields asymmetrically conveyed to promote healing processes and restore physiological balance within the human body [26].

It is based on a sophisticated system that generates carefully calibrated radio electric fields asymmetrically conveyed. These fields are designed to create an asymmetrical radio electric gradient within the body, with the specific purpose of optimizing cellular endogenous bioelectric activity (EBA). This optimization of EBA is essential for influencing cellular processes and biochemical reactions, ultimately leading to improved therapeutic effectiveness.

REAC induces the optimization of EBA, which in turn triggers various physiological mechanisms. These mechanisms include the activation of cellular signaling pathways, facilitation of ion transport, and stimulation of protein synthesis, among others. By modulating cellular activity through these mechanisms, REAC technology has the potential to enhance tissue repair and promote overall well-being.

The technology holds promising potential for enhancing neurobiological functions that are compromised in various pathological conditions. This innovative approach progressively promotes the processes of recovery, healing, and overall well-being [27].

By leveraging REAC technology, it becomes possible to address the underlying neurobiological dysfunctions associated with different pathologies. This includes promoting the restoration of disrupted neural processes, facilitating neuroplasticity, and optimizing neuronal communication. These mechanisms contribute to the overall improvement of neurological functions, leading to enhanced recovery and well-being [28,29].

Furthermore, the application of REAC technology has the potential to support the healing processes within the nervous system. By stimulating cellular and molecular responses, it can enhance tissue regeneration, mitigate inflammation, and optimize the neuroimmune system’s functioning. These factors collectively contribute to the overall improvement of neurological health and well-being [30].

One of the remarkable benefits of REAC therapies is their non-invasiveness and lack of side effects, rendering them a secure alternative for diverse patient populations.

Moreover, REAC treatments can be easily integrated into existing medical treatments, complementing traditional approaches and enhancing their outcomes.

#### 2.8.2. Neuro Postural Optimization (NPO)

REAC NPO is a non-invasive neuromodulation treatment that focuses on improving postural control and stability by modulating the neurobiological system. NPO recognizes the essential role of the central nervous system (CNS) in maintaining postural stability and movement control. By enhancing CNS function, NPO aims to optimize postural control and stability, which can provide relief from musculoskeletal condition, dysfunctional adaptations of muscle balance and postural distortions [31]. Detailed explanations of the administration methods have been extensively documented in previous publications [26,31].

#### 2.8.3. Neuro Psycho Physical Optimization and Neuro Psycho Physical Optimization Cervical Brachial

The specific REAC treatments employed in this study NPPO and NPPO-CB represent an advanced neuromodulation treatment that focuses on optimizing human performance by acknowledging the intricate relationship between the nervous system, psychological well-being, and physical body [29].

Clinical evidence supports the effectiveness of REAC NPPOs treatments in addressing various mood and behavioral disorders [32], including those associated with epigenetic modifications, such as autism spectrum disorder [28]. By considering the interplay between neurobiology, psychological factors, and physical functioning, REAC NPPOs offer a promising approach to managing and improving outcomes in these conditions.

Comprehensive descriptions of the administration methods have been extensively documented in prior publications [28].

#### 2.8.4. Neuromuscular Optimization (NMO)

NMO is the term used to encompass a set of therapeutic protocols for neurobiological modulation within the REAC technology aimed at optimizing muscle management, particularly the functional balance between agonist and antagonist muscles, in both pathological and dysfunctional conditions.

NMO therapies are performed by applying a special probe called asymmetric conveyer probe to the targeted areas, which is connected to the REAC device. Typically, multiple treatment cycles are implemented, varying based on the individual’s health or disease state. This approach aims to optimize muscle management and movement, enabling a gradual and progressive correction of neuro-psycho-motor alterations and the restoration of proper muscular and postural control [30].

### 2.9. Study Replicability

To ensure the replicability of the study, the REAC device (BENE mod 110-ASMED Srl Via Charta 77, 50018 Scandicci, Italy) used for administering the NPO, NPPOs, and NMO treatments employs fixed parameters that are set by the manufacturer. These parameters cannot be modified by the operators, thereby ensuring consistency across treatment administrations.

### 2.10. Statistical Analysis

For the statistical evaluation of the study, the G*Power (Universität Düsseldorf, Psychologie—HHU) and SPSS 22 software (IBM Corp., Armonk, NY, USA) were used.

To verify the normal distribution of the data, we used the Wilcoxon test and the sign test. Given the sample size, we consider the *p*-value of *p* < 0.50 to be statistically significant. The Analysis of Variance of Repeated Measures (ANOVA-RM) was performed on the scores of the TUG, HGST, and RPFS at four different time points: T0, T2, T3, and T4. A Bonferroni post-hoc test was used for a posteriori analysis when necessary. The effect size was expressed as η2 in all statistical analyses.

## 3. Results

The sample consisted of 5 men, mean age: 52.60, and 11 women, mean age: 56.70. One subject was unable to perform the last assessment due to COVID-19 infection. In this case, exclusively, the evaluations were considered up to T3 for statistical analysis.

### 3.1. Time Up and Go

**Figure 2:**
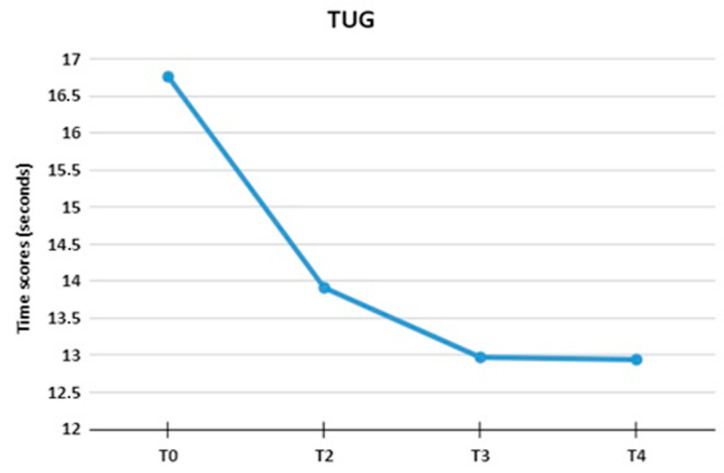
The figure shows the mean Time up and Go test time scores at T0, T2, T3, and T4.

When evaluating the TUG, a decrease in scores from T0 to T4 can be observed, as shown in Figure 2. This reduction was statistically significant (F (2.05, 30.88) = 15.83, *p* < 0.01; η2 = 0.51) when comparing the final time (T4) to the initial time (T0). A posteriori analysis (Bonferroni post-hoc) showed that there was a significant difference in the T0 moment (M = 16.76; SD = 6.98) in relation to the others: T2 (M = 13.91; SD = 4.95; *p* = 0.02), T3 (M = 12.97; SD = 4.97; *p* < 0.01), and T4 (M = 12.94; SD = 5.16; *p* < 0.01). The results also showed that the T2 assessment was not statistically different at T3 (*p* = 0.66) and T4 (*p* = 0.35). Furthermore, T3 did not differ statistically when compared to T4 (*p* = 1.00).

### 3.2. Handgrip Strength Test

**Figure 3:**
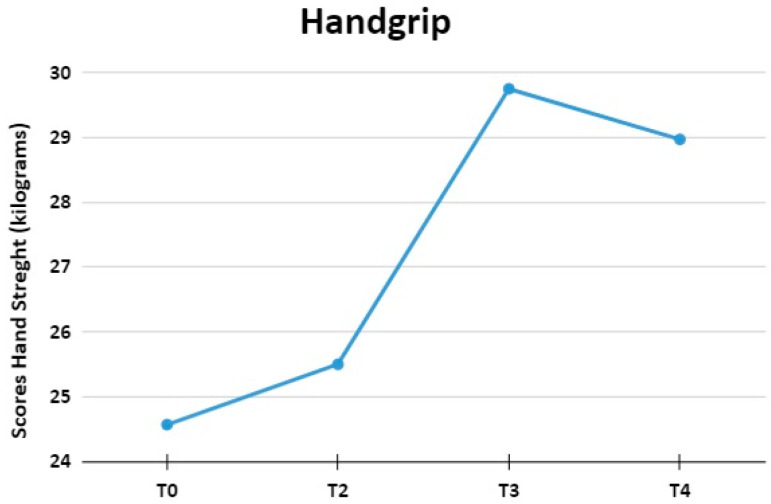
The figure shows the average scores of hand grip test at moments T0, T2, T3, and T4.

In the manual dynamometry evaluation, an Analysis of Variance of Repeated Measures (ANOVA-MR) was performed, with the objective of evaluating the scores of four moments: T0, T2, T3, and T4, as shown in Figure 3. The overall result showed that there were statistically significant differences in the mean strength of the assessments performed (F (1.28, 19.29) = 7.78, *p* < 0.01; η2 = 0.34).

A posteriori analysis (Bonferroni post-hoc) showed that there was a significant difference in the scores of the evaluated conditions. In this sense, there was a significant difference between T0 (M = 24.57; SD = 8.85) in relation to T4 (M = 28.97; SD = 10.03; *p* = 0.01) and also, from condition T2 (M = 25.50; SD = 9.10) in relation to T4 (*p* = 0.02). Regarding the other comparisons, there was no significant difference (*p* > 0.05).

### 3.3. Fatigue

**Figure 4:**
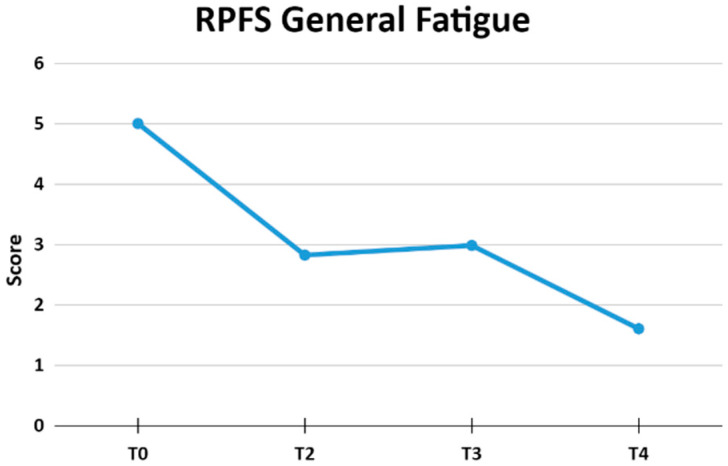
The figure shows the averages of the Revised Piper Fatigue Scale general fatigue scores at moments T0, T2, T3, and T4.

When evaluating the general fatigue through the RPFS, a statistically significant difference was observed when comparing the evaluation carried out at T0 in relation to moments T2, T3, and T4. The other comparisons performed did not show statistically significant differences, as shown in Table 1 (means and standard deviation) and Table 2 (Bonferroni *p*-values). Figure 4 shows the averages of the overall fatigue scores.

Post-hoc Bonferroni analysis showed the following differences in the scores of the RPF dimensions in the conditions evaluated, as shown in Table 2:

**Table 2:** Results of a posteriori comparisons between conditions in Revised Piper Fatigue Scale general fatigue. * Statistically significant (*p* < 0.05).

Revised Piper Fatigue Scale—General Fatigue (*p*-Values)				
	T0	T2	T3	T4
T0	-			
T2	<0.01 *	-		
T3	<0.01 *	>0.05	-	
T4	<0.01 *	>0.08	<0.10	-

**Figure 5:**
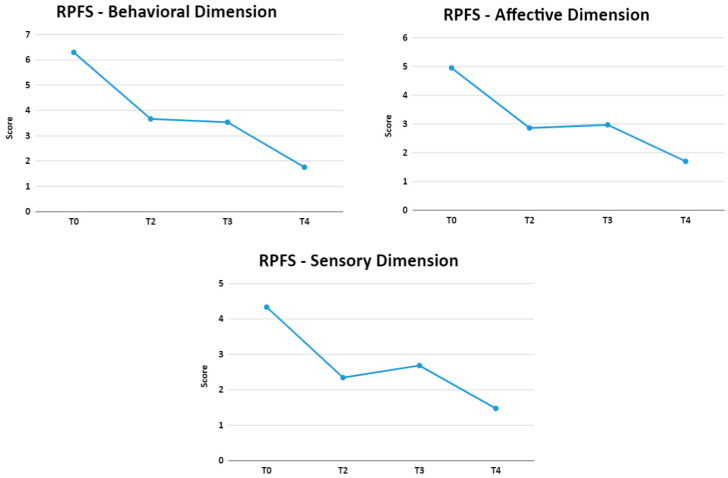
Means of Revised Piper Fatigue Scale dimensions at moments T0, T2, T3, and T4.

A Repeated Measures Analysis of Variance (ANOVA-MR) was performed with the objective of evaluating the RPFS scores according to the dimensions in four moments: T0, T2, T3, and T4, as shown in Figure 5. The result showed that there were statistically significant differences in the scores between the conditions described above in the dimensions behavioral (F (3, 45) = 20.42, *p* < 0.01; η2 = 0.57), affective (F (3, 45) = 29.08, *p* < 0.01; η2 = 0.45), sensory (F (3, 45) = 11.9, *p* < 0.01; η2 = 0.44), and fatigue (F (3, 45) = 31, 81, *p* < 0.01; η2 = 0.56).

The a posteriori analysis with post-hoc Bonferroni showed the following differences in the scores of the dimensions of the RPFS in the evaluated conditions, according to Table 3, Table 4 and Table 5 below:

## 4. Discussion

One of the primary reasons for the therapeutic difficulties in managing PPS is the lack of specific treatment options. Currently, there is no cure for PPS, and interventions primarily focus on symptom management and improving patients’ quality of life. The main symptoms of PPS are fatigue, pain, and new weakness. Rehabilitation programs for this population aim to minimize functional decline and maximize independence.

The intricate interplay between the physical, psychological, and epigenetic aspects of PPS poses additional challenges in providing the best and most appropriate therapies. In this context, the results found in the present study are of great importance since improvements in mobility and functional performance, handgrip strength, and general fatigue and in the dimensions assessed (affective, sensory, and behavioral) were demonstrated when compared to the initial assessment (T0) with the final assessment (T4).

Considering the mobility and functional performance, the participants presented improvement when comparing T0 on TUG to the other evaluations (T2, T3, and T4). The test is commonly used to measure mobility and functional performance in people with PS and PPS [33,34]. The initial treatment approach in this study was the REAC NPO, which aims to optimize neuro psychomotor strategies through functional and epigenetic adaptive processes [26,31]. It was hypothesized that this treatment would predominantly affect the motor aspect, leading to an anticipated improvement in the TUG test (*p* < 0.01).

In fact, when evaluating the results presented, an improvement in the performance of the TUG was obtained, that is, a reduction in the time measured by the scale. This result may infer an improvement in their gait functionality, safety, and efficiency. Furthermore, a progressive improvement in the score was presented in the second reevaluation (T3), demonstrating an improvement in the gait rhythm after performing the NPPO and NPPO-CB, despite not showing a statistically significant difference. However, after applying the NMO protocol, there was stability in the TUG score (T3–T4).

This result is important as individuals with PPS may experience a decline in their capacity to swiftly navigate obstacles while walking. This can contribute to a perceived decrease in balance and an increased sense of apprehension regarding the risk of falling [35]. Falling is common in people with PS and PPS, totaling almost 70% incidence, and they have a four times higher fall rate compared to the elderly [36,37]. This constitutes an aggravating factor since one-third of those who fall suffer fractures due to bone fragility in the limb involved by poliomyelitis. Patients with PPS may develop lasting musculoskeletal deformities or contractures as a way to improve their walking speed and balance. Among the main reasons for falls are: muscle weakness (especially knee extensor muscles), fatigue, joint and muscle pain, reduced gait performance, dynamic balance deficit, and fear of falling [36,37].

To decrease this incidence of falling, many strategies are adopted, such as: walking aids, wheelchair, motorized wheelchair, adapting the patient’s home environment, and exercises to control balance and strength [38], but most post-polio patients saw a gradual decrease in their physical mobility over the course of a decade [37]. REAC treatments have been shown to be effective in various neurological movement disorders [30]. In this study, NPO was able to optimize gait functionality in individuals diagnosed with PPS. This underscores the potential of addressing the dysfunctional aspect of the condition using this therapeutic approach and shows that REAC technology may be a potential treatment to reduce falls in this population.

It was demonstrated in an fMRI study that a 250-millisecond REAC NPO pulse was capable of producing changes in brain activity that were apparent 40 min after the stimulus [26]. Furthermore, there was an improvement in patellar symmetry (functional dysmetria) when asked to sit up from the lying position. The authors suggest that these changes occurred due to changes in brain activity, suggesting more efficient motor control and motor strategy [26].

An important finding was that the application of the REAC protocols that were part of the present study resulted in an increase in handgrip strength when comparing T0 with T4 (protocols NPO, NPPO+NPPO-CB, and NMO). This result is of great importance since it is well established that handgrip exhibits a robust correlation with an individual’s overall muscle strength [26]. Furthermore, according to [39], handgrip strength demonstrates predictive validity for cognitive decline, mobility impairment, changes in functional status, and mortality in older adults living in community settings.

These data gain even more significance when we take into account that the sample population consisted of individuals over the age of 60, with an average age of 52.6 for women and 56.7 for men. Research on the degradation of muscle strength reveals an annual decline of 1% in the general population after the age of 50, while individuals with PPS experience a decline of 2% per year [40,41]. Despite this, participants showed a statistically significant improvement in handgrip strength.

Many studies correlated handgrip strength to functionality [42,43,44], and it can be speculated that low strength is associated with a worsening in the performance of daily live activities.

Another aspect evaluated in the present work was fatigue, which is one of the most frequent, incapacitating, and debilitating symptoms and causes the greatest interference in the everyday activities of the patients [6,45]. The cause of the symptom is of a multifactorial nature and can be divided into two components: peripheral (muscular) fatigue and central (general or simultaneous) fatigue [46]. For this measurement, the RPFS was used, which revealed a statistically significant reduction in overall fatigue scores, from a moderate overall fatigue level (5 ± 2.08) to a mild fatigue level (1.6 ± 1.8) at T4 (*p* < 0.0001). These results demonstrate the effectiveness of REAC neurobiological modulation in people with PPS, even though fatigue is a complex and debilitating symptom of this neurodegenerative disease. The RPFS presents three main topics: affective/sensorial, psychological, and behavioral.

Interestingly, it was believed that the application of NPO (T1), which aims to optimize neuro psychomotor strategies through functional and epigenetic adaptive processes [26,31], would predominantly affect the physical aspects evaluated, leading to a more significant improvement in TUG and HGST, however, improvement was observed in all aspects of the instruments used, even observing the dimensions of the RPFS.

The affective dimension seeks to identify the interpretation or meaning attributed to fatigue. A statistically significant improvement (*p* < 0.0001) was observed in the fatigue indices of this dimension when comparing the moment T0 (Moderate; 4.95 ± 2.7) with T4 (Light; 1.7 ± 2.29). Throughout the treatment, a slight fluctuation of the symptom was observed with a small increase in the symptom from T2 to T3 (Post-NPPO). When evaluating the classification indicated by the instrument, it did not change. The same results were found in the sensory/psychological dimension, reporting moderate initial fatigue (T0 = 4.33 ± 2.56) evolving to mild fatigue in the other phases of the study even with small fluctuations (T2 = 2.34 ± 1.76; T3 = 2.68 ± 2.02, and T4 = 1.47 ± 1.67). This dimension brings together self-perception, emotional and cognitive components to the presence of fatigue. These findings suggest that enhancing neuro psychomotor strategies, even without the explicit involvement of the patient, can result in substantial improvements in the investigated neuropsychic domains assessed by the RPFS. The fluctuation in fatigue rates, with a slight increase, occurred in the treatments of NPPOs (moments T2 to T3) in the affective and sensory/psychological dimensions despite them having been administered precisely to treat mood and behavioral disorders. It was expected that these treatments would produce more noticeable results on the RPFS, but statistically significant improvements were observed mainly in motor aspects, as demonstrated above. These findings suggest that, despite the administration of treatments with NPPOs, known for their broad and demonstrated efficacy, improvement in several mood and behavioral disorders [28,29,32] that influence the clinical and symptomatic progression of patients with PPS may not be immediately noticed. Unconsciously, this improvement may initially result in emotional instability, possibly due to the disruption of long-standing dysfunctional neuro psycho adaptive patterns.

On the other hand, the behavioral dimension showed progressive improvement in all evaluations. This was the dimension that presented higher fatigue indices (Intense fatigue T0: 6.29 ± 1.76) and demonstrated the greatest reduction at the end of the study (Mild fatigue T4: 1.75 ± 1.97). It represents functional capacity-related components that can be impaired by fatigue, such as personal issues, social activities, and sexual relationships. It is interesting to note that REAC did not propose any physical changes in these patients; the deficiency remains present, but their behavior towards these deficiencies is modified.

Treatments targeting NPPOs have been administered to treat mood and behavioral disorders [28,29]. It was expected that these treatments would produce more noticeable results on the RPFS. However, a slight change or maintenance of fatigue indices was observed in all dimensions evaluated.

Likewise, the NMO protocol is specifically designed as an advanced way to recover the correct facilitation and inhibition mechanisms, both ipsilateral and contralateral to the muscle groups. However, it did not have statistically positive effects when evaluated separately, despite presenting maintenance of the values obtained in strength assessments and gait function (TUG and HGST) in addition to improvement.

On the other hand, the results obtained in this study point to an interaction between the treatments since the NMO protocol promoted a reduction in fatigue and the application of the neuropsychic aspects protocols (NPPO and NPPO-CB) played an important action on motor control items and functionality (TUG and HGST). Corroborating the above [47], report that motor performance and emotions have a bidirectional relationship. That is, an altered emotional state is associated with changes in motor performance, as well as changes in movement production can trigger mood changes, including symptoms of depression and anxiety [47].

All this, when applied to the reality of patients with PPS, helps us better understand how neurobiological modulation treatments, such as REAC NPO, NPPOs, and NMO, can represent a new therapeutic strategy to assist PPS patients in both physical and psychological aspects.

It is worth mentioning that the population studied consists of people who suffer from a chronic degenerative disease in which there is a series of adaptations, whether physical, emotional and/or behavioral, already installed. This reinforces the importance of the data found in such a short intervention period of the protocols used in the present study. Furthermore, despite the search for other therapeutic strategies to manage fatigue and improve functional performance, results as significant as those demonstrated in the present study were not found for this population.

The limitations of this study lie in its open nature, with a relatively small but still significant sample, and patients who, despite sharing the diagnosis of PPS, present varying degrees of disability. Therefore, it is necessary to carry out further research in order to verify whether specific treatments using REAC neurobiological modulation treatments can represent a valid strategy for improving fatigue, functional performance and, consequently, quality of life in patients with PPS.

## 5. Conclusions

Given the data presented, it can be inferred that the intervention through the REAC NPO, NPPOs, and NMO treatments demonstrated positive effects in improving the fatigue index, functional performance of gait, and handgrip strength. Furthermore, the use solely of the NPO protocol proved to be a good therapy for improving the fatigue index and functional gait performance. The results also suggest the need for new studies with longer intervention times, as well as an expansion of the sample and a different design (clinical trial) in order to better investigate the effects of the interventions.

## Figures and Tables

**Table 1:** Means and Standard Deviation (SD) of the Revised Piper Fatigue Scale dimensions.

Revised Piper Fatigue Scale—Fatigue				
Conditions (Mean ± SD)	Behavioral Dimension	Affective Dimension	Sensory Dimension	Fatigue
T0	6.29 ± 1.76	4.95 ± 2.70	4.33 ± 2.56	5.00 ± 2.08
T2	3.66 ± 2.37	2.86 ± 2.20	2.34 ± 1.76	2.82 ± 1.93
T3	3.53 ± 2.95	2.97 ± 3.21	2.68 ± 2.02	2.98 ± 2.35
T4	1.75 ± 1.97	1.70 ± 2.29	1.47 ± 1.67	1.60 ± 1.80

**Table 3:** Results of a posteriori comparisons between the different moments in the behavioral dimension of the Revised Piper Fatigue Scale. * Statistically significant (*p* < 0.05).

Revised Piper Fatigue Scale—Behavioral Dimension (*p*-Values)				
Moments	T0	T2	T3	T4
T0	-			
T2	<0.01 *	-		
T3	<0.01 *	>0.05	-	
T4	<0.01 *	0.01 *	<0.07	-

**Table 4:** Results of a posteriori comparisons between different moments in the affective dimension of the Revised Piper Fatigue Scale. * Statistically significant (*p* < 0.05).

Revised Piper Fatigue Scale—Affective Dimension (*p*-Values)				
Moments	T0	T2	T3	T4
T0	-			
T2	<0.01 *	-		
T3	<0.05 *	>0.05	-	
T4	<0.01 *	>0.05	>0.05	-

**Table 5:** Results of a posteriori comparisons between the different moments in the sensory dimension of the Revised Piper Fatigue Scale. * Statistically significant (*p* < 0.05).

Revised Piper Fatigue Scale—Sensory Dimension (*p*-Values)				
Moments	T0	T2	T3	T4
T0	-			
T2	<0.01 *	-		
T3	0.01 *	>0.05	-	
T4	<0.01 *	>0.05	>0.05	-

## Data Availability

Data is contained within the article.
